# Evaluation of the Effects of a Combination of Japanese Honey and Hydrocolloid Dressing on Cutaneous Wound Healing in Male Mice

**DOI:** 10.1155/2015/910605

**Published:** 2015-04-07

**Authors:** Kanae Mukai, Miki Koike, Saki Nakamura, Yuka Kawaguchi, Fumika Katagiri, Saki Nojiri, Yuki Yamada, Eri Miyajima, Mayuko Matsumoto, Emi Komatsu, Yukari Nakajima, Tamae Urai, Naoko Murakado, Toshio Nakatani

**Affiliations:** ^1^Department of Clinical Nursing, Graduate Course of Nursing Science, Division of Health Sciences, Graduate School of Medical Sciences, Kanazawa University, 5-11-80 Kodatsuno, Kanazawa 920-0942, Japan; ^2^Department of Nursing, School of Health Sciences, Kanazawa University, 5-11-80 Kodatsuno, Kanazawa 920-0942, Japan; ^3^School of Nursing, Kanazawa Medical University, 1-1 Daigaku, Uchinada, Kahoku 920-0293, Japan; ^4^Faculty of Health Sciences, Institute of Medical, Pharmaceutical and Health Sciences, Kanazawa University, 5-11-80 Kodatsuno, Kanazawa 920-0942, Japan

## Abstract

The aim of this study was to evaluate the effect of the combined use of Japanese honey and hydrocolloid dressing (HCD) on cutaneous wound healing. Mice were divided into four groups: the Acacia (Japan) + HCD, Manuka (New Zealand) + HCD, Chinese milk vetch (Japan) + HCD, and HCD (control) groups. The mice received two full-thickness wounds. The wounds of the HCD group were covered with HCD, whereas those of the other groups were treated with 0.1 mL of the relevant type of honey, before being covered with HCD. Wound area was significantly smaller in the HCD group than in the Acacia + HCD and Manuka + HCD groups on day 13 and days 8–14, respectively. Moreover, compared with the HCD group, reepithelialization was delayed in the Acacia + HCD group and reepithelialization and collagen deposition were delayed in the Chinese milk vetch + HCD and Manuka + HCD groups. These results indicate that the combined use of Japanese honey and HCD does not promote cutaneous wound healing compared with the use of HCD alone. Thus, this method is probably not useful for promoting healing.

## 1. Introduction

Honey is a sweet, sticky substance that is produced by bees following the collection of nectar and honeydew [1]. Its mean composition is as follows: 17.1% water; 82.4% total carbohydrates; and 0.5% proteins, amino acids, vitamins, and minerals [2]. The main carbohydrates found in honey are fructose (38.5%) and glucose (31%), with maltose, sucrose, and other sugars making up the remaining 12.9% [2]. Honey has been used to treat wounds since ancient times [3], and Russian soldiers used honey to promote wound healing in World War I [4]. Honey is considered to be useful for treating wounds due to its antibacterial, anti-inflammatory, and immunostimulatory effects, which are related to its hygroscopicity, sugar content, and the fact that it contains hydrogen peroxide [5].

Initially, researchers only used pure, nonboiled commercial honey when evaluating the effects of honey on cutaneous wound healing [4, 6, 7]. However, it has subsequently been demonstrated that different types of honey exhibit substantially different activities, which is partly dependent on their source [8]. Therefore, numerous types of honey from around the world have had their effects on cutaneous wound healing evaluated. For example, studies of Manuka honey [9–13] and mixed pasture honey [9, 10] from New Zealand, jelly bush honey [10] from Australia, Acacia honey [14] from Pakistan, Gelam honey [8] and Tualang honey [13] from Malaysia, and Indonesian honey [15] have been performed. On the other hand, to the best of our knowledge, only Ranzato et al. have reported on the effects of Japanese honey (Yamada Apiculture Center, Inc., Okayama, Japan), and their study had an in vitro design [16]. Therefore, we previously macroscopically and microscopically investigated the in vivo effects of three types of Japanese honey, Acacia honey, buckwheat flour honey, and Chinese milk vetch honey, as topical therapies for promoting cutaneous wound healing [17]. As a result, we found that although Ranzato et al. reported that honey-driven wound repair involves the activation of keratinocyte reepithelialization in vitro, the application of Japanese honey did not promote cutaneous wound in vivo healing compared with that seen in mice treated with hydrocolloid dressings (HCD). However, our previous results also showed that during the inflammatory phase the wound area ratio (see below) and the number of macrophages were significantly decreased in mice treated with Japanese honey compared with those seen in mice treated with HCD [17]. Based on these findings, we considered that the combined use of Japanese honey and HCD, with honey being applied to the wound followed by a HCD, might promote cutaneous wound healing to a greater extent than the use of HCD alone. Specifically, we considered that Japanese honey would reduce the inflammatory response induced at the wound site and HCD would promote reepithelialization, collagen deposition, and wound contraction.

Therefore, in this study, we investigate the effects of the combined use of Japanese honey and HCD on cutaneous wound healing in young male mice. We hypothesized that cutaneous wound healing would be promoted in young male mice treated with Japanese honey and HCD compared with that seen in young male mice treated with HCD alone.

## 2. Materials and Methods

### 2.1. Animals

Seventy-six BALB/cCrSlc, 8-week-old, male mice (Sankyo Lab Service Corporation, Inc., Toyama, Japan) with a mean weight of 24.2 ± 0.7 g were used in this study. The mice were caged individually in an air-conditioned room at 25.0 ± 2.0°C, in which the lights were switched on from 08:45 to 20:45 hours and were given ad libitum access to water and chow. All of the animal experiments conducted in this study were reviewed and approved by Kanazawa University Animal Experiment Committee and were carried out in accordance with the Guidelines for the Care and Use of Laboratory Animals of Kanazawa University, Japan (AP-132886).

### 2.2. Honey

We used three types of honey within 1 year of purchase: Manuka (*Leptospermum scoparium*) honey (UMF 15+) from New Zealand and Acacia (*Robinia pseudoacacia*) honey and Chinese milk vetch (*Astragalus sinicus*) honey (Yamada Bee Farm, Okayama, Japan) from Japan. The Manuka honey was composed of 17.3% water, 82.1% total carbohydrates, 0.4% protein, and 0.2% ash and had an energy density of 330 kcal/100 g (Foundation of Food Analysis Technology Center SUNATEC, Mie, Japan). The Acacia honey was composed of 17.2% water, 82.5% total carbohydrates, 0.2% protein, and 0.1% lipids and had an energy density of 332 kcal/100 g (Foundation of Food Analysis Technology Center SUNATEC, Mie, Japan). The Chinese milk vetch honey was composed of 17.0% water, 82.7% total carbohydrates, 0.2% protein, and 0.1% lipids and had an energy density of 333 kcal/100 g (Foundation of Food Analysis Technology Center SUNATEC, Mie, Japan). The composition of three types of honey was almost the same. The Acacia honey and Chinese milk vetch honey were selected based on the results of our previous study [17], and the Manuka honey was selected as a positive control because it is probably the most well known of the honeys that are used as topical therapies.

### 2.3. Wounding

**Figure 1:**
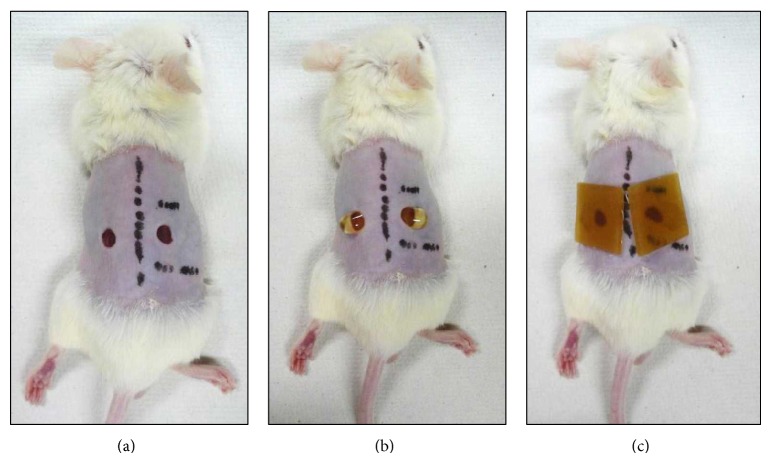
Wound treatment. (a) Two circular full-thickness skin wounds (4 mm in diameter). (b) Each of the experimental wounds was treated with 0.1 mL of honey. (c) All wounds were covered with HCD. In the experimental groups, the wounds were treated with 0.1 mL of honey (b), before being covered with HCD (c). On the other hand, the wounds of the control group were only covered with HCD (c).

The mice were anesthetized via the intraperitoneal (IP) injection of pentobarbital sodium (0.05 mg/g weight), and their dorsal hair was shaved off. Then, under anesthesia, two circular full-thickness skin wounds (4 mm in diameter), which included the panniculus carnosus muscle, were made on both sides of the dorsum using a Kai sterile disposable biopsy punch (Kai Industries Co. Ltd., Gifu, Japan) (Figure 1(a)). The mice were divided into four groups: the Acacia + HCD group, the Manuka + HCD group, the Chinese milk vetch + HCD group, and the HCD group (control group). In the experimental groups, each wound was treated with 0.1 mL of honey, covered with a HCD (Tegaderm; 3M Health Care, Tokyo, Japan), and then wrapped with sticky bandages (Meshpore tape; Nichiban, Tokyo, Japan) (Figures 1(b) and 1(c)). The HCD were changed and all wounds were treated with honey every day. On the other hand, the wounds of the control group were only covered with HCD and then wrapped with sticky bandages, which were changed every day (Figure 1(c)).

### 2.4. Macroscopic Examination

The day when the wounds were made was designated as day 0, and wound healing was observed from days 0 to 14. The edges of the wounds were traced on polypropylene sheets, and photographs of the wounds were taken every day. The traces on the polypropylene sheets were then captured with a scanner and transferred to a personal computer using Adobe Photoshop Elements 7.0 (Adobe System Inc., Tokyo, Japan). The area of each wound was calculated using the image analysis software Scion Image Beta 4.02 (Scion Corporation, Frederick, Maryland, USA). At each time point, wound area is shown as a proportion of the wound area on day 0 (the wound area ratio).

### 2.5. Plasma TNF-*α* and TGF-*β* Levels

Mice were euthanized via the IP injection of pentobarbital sodium on days 3 or 7. Plasma was prepared from each mouse's blood, which was obtained through cardiac puncture, and frozen until the time of the assay. The plasma levels of tumor necrosis factor (TNF-) *α* and transforming growth factor (TGF-) *β* were determined using enzyme-linked immunosorbent assays (ELISA) (R&D Systems, Tokyo, Japan), according to the manufacturer's guidelines.

### 2.6. Histological Procedure and Immunohistological Staining

Mice were euthanized via the IP injection of a lethal dose of pentobarbital sodium on days 3, 7, 11, or 14 after wounding. The wound and the surrounding intact skin were harvested, and each sample of wound tissue and the surrounding intact skin was bisected at the center of the wound. The wound samples were stapled onto polypropylene sheets to prevent overcontraction, before being fixed in 4% paraformaldehyde for 18 hours. The samples were then dehydrated in a graded alcohol series, cleaned in xylene, and embedded in paraffin, before 5-*μ*m serial paraffin sections were prepared. At least 6 serial sections from near to the center of the wound were obtained per wound and stained according to the following methods [17]. Five-*μ*m thick sections were subjected to hematoxylin-eosin (HE) or azan staining or were immunohistologically stained with anti-neutrophil antibody to detect neutrophils (1 : 100; Abcam Japan, Tokyo, Japan), anti-Mac-3 antibody to detect macrophages (1 : 100; BD Pharmingen, Tokyo, Japan), or anti-*α*-smooth muscle actin (*α*-SMA) antibody to detect myofibroblasts (1 : 300; Abcam Japan, Tokyo, Japan). Negative control slides were obtained by omitting each primary antibody.

### 2.7. Microscopic Observations

**Figure 2:**
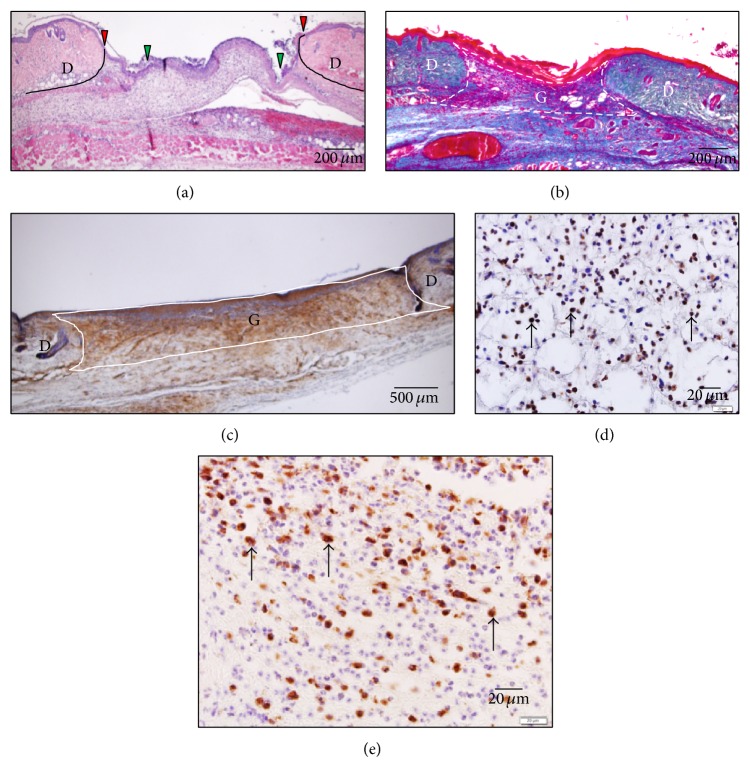
Macroscopic observations. (a) HE staining on day 3. The red arrows indicate the wound edges, and the green arrows indicate the edge of the new epithelium (bar, 200 *μ*m). (b) Azan staining on day 7. The white box indicates the wound area; that is, it surrounds both wound edges, the surface of the wound, and the bottom of the wound (bar, 200 *μ*m). (c) Immunohistological staining of myofibroblasts on day 14. Myofibroblasts were stained with anti-*α* SMA antibody. The white box indicates the wound area; that is, it surrounds both wound edges, the surface of the wound, and the bottom of the wound (bar, 500 *μ*m). (d) Immunohistological staining of neutrophils on day 3. Neutrophils (arrows) were stained with anti-neutrophil antibody (bar, 20 *μ*m). (e) Immunohistological staining of macrophages on day 3. Macrophages (arrows) were stained with anti-Mac-3 antibody (bar, 20 *μ*m). D: dermis; G: granulation tissue.

Images of the wounds were obtained using a digital microscopic camera (DP2-BSW, Olympus, Japan) and then imported onto a computer. Assessments of reepithelialization were performed using the software DP2-BSW Olympus. In these assessments, the distance between the edges of the wound and the length of the new epithelium were measured, and then the latter was divided by the former (length of new epithelium/total wound length; the reepithelialization ratio) (Figure 2(a)). Evaluations of collagen (which was stained blue) deposition (collagen pixels/total wound pixels; the collagen fiber ratio) and the number of myofibroblasts, which were stained brown, (myofibroblast pixels/total wound pixels; the myofibroblast ratio) were performed using Adobe Photoshop Elements 7.0 as follows: the wound area, that is, both wound edges, the surface of the wound, and the bottom of the wound, including the panniculus carnosus muscle, was first selected, and then the number of pixels in the selected area (the wound area) was calculated. Next, assessments of collagen deposition or the number of myofibroblasts were performed by counting the number of blue or brown pixels. Finally, the number of blue or brown pixels was divided by the total number of pixels within the wound area (Figures 2(b) and 2(c)). To analyze the numbers of neutrophils and macrophages in granulation tissue, the number of positively stained cells was counted under a light microscope using a ×40 objective lens in three regions of granulation tissue (two sites near the two wound edges and a site in the center of the granulation tissue). The areas of these three sites were calculated using the image analysis software Image J (National Institutes of Health, Bethesda, Maryland, USA), and the total number of neutrophils or macrophages found at the three sites was divided by the total area of the three sites (Figures 2(d) and 2(e)).

### 2.8. Statistical Analysis

Data are expressed as mean ± SD values and were analyzed using JMP 8.0.1 (SAS Institute Inc., Cary, NC, USA). The significance of differences was assessed using ANOVA or the Tukey-Kramer multiple comparisons test. Differences were considered significant at *P* < 0.05.

## 3. Results

### 3.1. Wound Area

In all groups, the HCD absorbed so much of the exudate that they expanded. In the experimental groups, the HCD were easily removed. On the other hand, in the HCD group (control group) the HCD were difficult to remove because they stuck to the wound or surrounding skin.

The wound area ratio was calculated on days 1 to 14. In the Acacia + HCD group, wound area did not increase until day 6, and then it decreased rapidly until day 7, after which it decreased gradually until day 14 (day 14 wound area ratio: 0.36 ± 0.07). In the Chinese milk vetch + HCD and Manuka + HCD groups, wound area increased for 6 days. In the Chinese milk vetch + HCD group, it then decreased rapidly until day 7, before decreasing gradually until day 14 (day 14 wound area ratio: 0.31 ± 0.24). In the Manuka + HCD group, wound area also decreased rapidly until day 7, but then increased again, before gradually decreasing until day 14 (day 14 wound area ratio: 0.56 ± 0.23). On the other hand, in the HCD group wound area increased for 4 days and then decreased rapidly until day 10, after which it decreased slowly until day 14 (day 14 wound area ratio: 0.16 ± 0.13).

**Figure 3:**
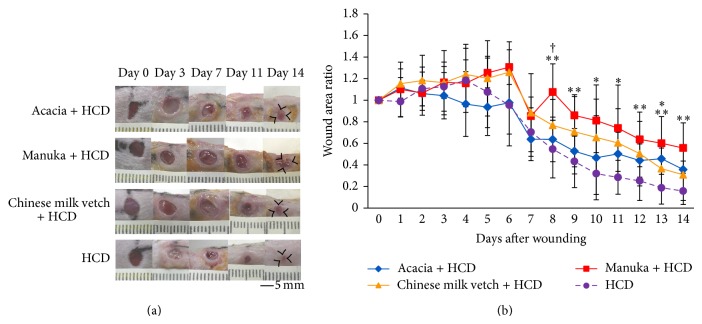
Macroscopic wound healing. (a) Wounds of four mm in diameter were inflicted, and healing was recorded photographically (bar, 5 mm). (b) The wound area ratios observed at each time point. Data are expressed as mean ± SD values. *n* = 6–10, ^*^ *P* < 0.05 and ^**^ *P* < 0.01: HCD group versus honey + HCD groups, ^†^ *P* < 0.05: Acacia + HCD group versus Manuka + HCD group (ANOVA, Tukey-Kramer).

The wound area ratio did not differ significantly among the groups on days 1–7, that is, during the early and late inflammatory phases. On the other hand, the wound area ratio of the Acacia + HCD group was significantly greater than that of the HCD group on day 13 (*P* = 0.0440). In addition, the wound area ratio of the Manuka + HCD group was significantly greater than that of the HCD group on days 8–14 (*P* = 0.0012, 0.0082, 0.0172, 0.0122, 0.0093, 0.0007, and 0.0006, resp.). Moreover, the wound area ratio of the Manuka + HCD group was also significantly greater than that of the Acacia + HCD group on day 8 (*P* = 0.0195) (Figure 3(b)).

### 3.2. Neutrophils, Macrophages, and Plasma TNF-*α* Level

**Figure 4:**
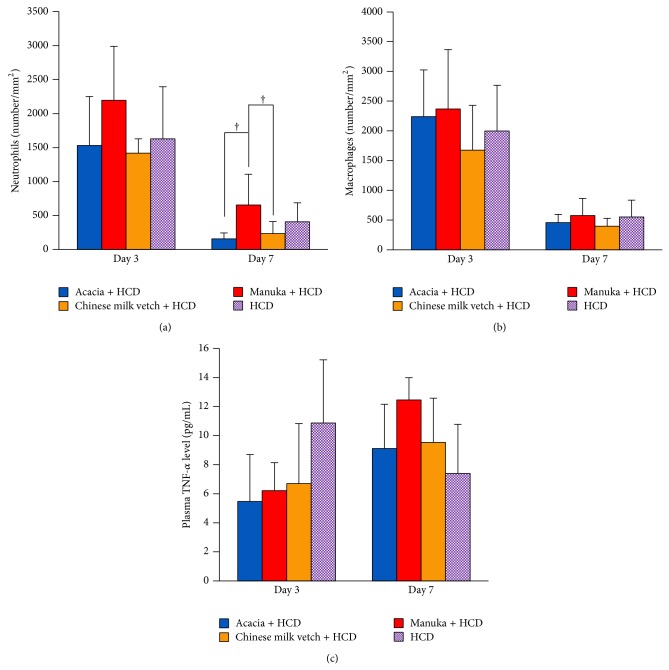
Neutrophils, macrophages, and plasma TNF-*α* level. (a) The number of neutrophils per mm^2^, (b) the number of macrophages per mm^2^, and (c) the systemic TNF-*α* level (pg/mL) are shown. Data are expressed as mean ± SD values. *n* = 4–8 for each group; ^†^ *P* < 0.05: Acacia + HCD group or Chinese milk vetch + HCD group versus Manuka + HCD group (ANOVA, Tukey-Kramer).

On day 3, numerous neutrophils were observed in the wounds of all groups, and they decreased in number until day 7. On day 7, a significantly greater number of neutrophils was detected in the Manuka + HCD group than in the Japanese honey + HCD groups (the Acacia + HCD and Chinese milk vetch + HCD groups) (*P* = 0.0152 and 0.0484, resp.). However, there were no significant differences in the number of neutrophils between the HCD and experimental groups on days 3 or 7 (Figure 4(a)).

On day 3, numerous macrophages were also observed in the wounds of all groups, and they decreased in number until day 7. There were no significant differences in the number of macrophages among the groups on days 3 or 7 (Figure 4(b)).

The plasma TNF-*α* level of the HCD group peaked on day 3 and decreased until day 7, whereas the plasma TNF-*α* levels of the experimental groups were low on day 3 and peaked on day 7. However, there were no significant differences in the plasma TNF-*α* levels of the four groups on days 3 or 7 (Figure 4(c)).

### 3.3. Reepithelialization, Collagen Deposition, Wound Contraction, and the Plasma TGF-*β* Level

**Figure 5:**
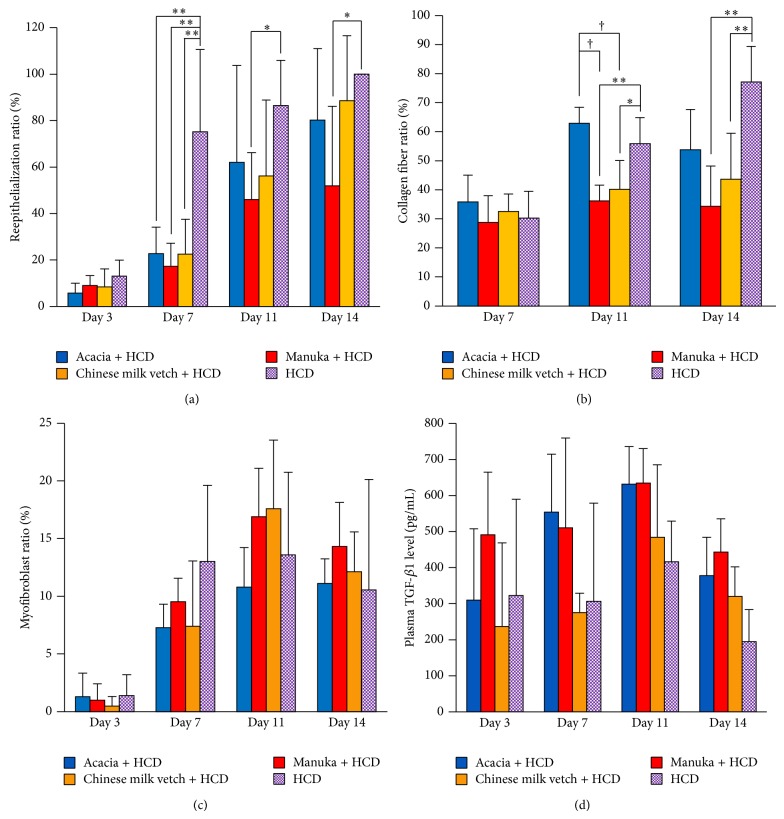
Reepithelialization, collagen deposition, wound contraction, and plasma TGF-*β*1 level. (a) The reepithelialization ratio (%), (b) the collagen fiber ratio (%), (c) the myofibroblast ratio (%), and (d) the systemic TGF-*β*1 level (pg/mL) are shown. Data are expressed as mean ± SD values. *n* = 4–8 for each group; ^*^ *P* < 0.05 and ^**^ *P* < 0.01: HCD group versus honey + HCD group, ^†^ *P* < 0.05: Acacia + HCD group versus Chinese milk vetch + HCD group or Manuka + HCD group (ANOVA, Tukey-Kramer).

On day 3, new epithelial tissue was seen at the wound edges in all groups. The new epithelial tissue gradually covered the wound surface as wound healing progressed. By day 14, the new epithelial tissue had completely covered the wound surface in the HCD group, but it did not in the experimental groups. On day 7, the reepithelialization ratio of the HCD group was significantly greater than those of the Acacia + HCD and Chinese milk vetch + HCD groups (*P* < 0.000 and <0.000, resp.). In addition, it was significantly greater than that seen in the Manuka + HCD group on days 7–14 (*P* < 0.000, *P* = 0.020 and 0.017, resp.). However, there were no significant differences in the reepithelialization ratio among the experimental groups on days 3–14 (Figure 5(a)).

Collagen deposition increased as wound healing progressed in all groups. The collagen fiber ratio of the HCD group was significantly greater than those of the Manuka + HCD and Chinese milk vetch + HCD groups on days 11 and 14 (HCD versus Manuka + HCD: *P* = 0.006 and *P* < 0.000, resp.; HCD versus Chinese milk vetch + HCD: *P* = 0.035 and 0.005, resp.). Moreover, the collagen fiber ratio of the Acacia + HCD group was significantly greater than those of the Manuka + HCD and Chinese milk vetch +HCD groups on day 11 (*P* < 0.000 and *P* = 0.002, resp.) (Figure 5(b)).

On day 3, a few myofibroblasts were observed at the wound sites in all groups. By day 11, many myofibroblasts were present in the granulation tissue. There were no significant differences in the number of myofibroblasts among the four groups on days 3–14 (Figure 5(c)).

In all groups, the plasma TGF-*β* level gradually increased, peaked on day 11, and then decreased until day 14. There were no significant differences in the plasma TGF-*β* level among the four groups on days 3–14 (Figure 5(d)).

## 4. Discussion

Previously, we compared the effects of three types of Japanese honey (Acacia, Chinese milk vetch, and buckwheat honey) on cutaneous wound healing in young male mice with those of HCD treatment [17]. As a result, we found that the wound area of the HCD group increased during the inflammatory phase and then rapidly decreased and that the wounds in this group had healed (although they exhibited scarring) by day 14. On the other hand, in the Japanese honey treatment groups wound area decreased during the inflammatory phase but then repeated episodes of expansion and reduction occurred. Moreover, the wound area ratio of the HCD group was significantly greater than those of the Acacia and Chinese milk vetch honey groups on days 1–8 and was significantly greater than that of the buckwheat honey group on days 1–5. In addition, the number of macrophages in the HCD group was significantly greater than those seen in the Japanese honey treatment groups on days 3 and 7. Furthermore, the wound area ratio did not differ significantly between the HCD group and the Japanese honey treatment groups on days 9–14, and the HCD group demonstrated a significantly greater reepithelialization ratio than the Japanese honey treatment groups on days 7–14. These results indicate that the application of Japanese honey reduced the inflammatory responses induced at the wound sites but did not promote cutaneous wound healing to a greater degree than HCD. Therefore, we considered that the combined use of Japanese honey and HCD might promote cutaneous wound healing to a greater extent than the use of HCD alone; however, the present study demonstrated that this was not the case. In the inflammatory phase, wound area increased in the HCD and Japanese honey (Acacia and Chinese milk vetch) + HCD groups, and the wound area ratio, the numbers of neutrophils and macrophages, and the plasma TNF-*α* level did not differ significantly between the HCD and Japanese honey + HCD groups. These results show that the combined use of Japanese honey and HCD did not reduce the inflammatory responses induced at the wound sites compared with the application of Japanese honey alone. The reason for this remains unclear. However, the anti-inflammatory effects of honey might be inhibited by the concomitant use of HCD. After the inflammatory phase, wound area decreased in both the HCD and Japanese honey + HCD groups. However, on day 14 the wound area ratio was 0.16 ± 0.13 in the HCD group, 0.36 ± 0.07 in the Acacia + HCD group, and 0.31 ± 0.24 in the Chinese milk vetch + HCD group. The wound area ratio of the HCD group was significantly lower than that of the Acacia + HCD group on day 13, the reepithelialization ratio of the HCD group was significantly greater than those of the Japanese honey + HCD groups on day 7, and the collagen fiber ratio of the HCD group was significantly greater than that of the Chinese milk vetch + HCD group on days 11 and 14. These results indicate that the combined use of Japanese honey and HCD did not promote cutaneous wound healing compared with the use of HCD alone. Therefore, the combined use of Japanese honey and HCD is probably not a useful method for promoting cutaneous wound healing.

It has been reported that Manuka honey, which is the most well known of all of the honeys used for wound treatment, promotes cutaneous wound healing. Gethin and Cowman found that treatment with Manuka honey was associated with an increased incidence of healing, effective desloughing, and a lower incidence of infection than standard hydrogel therapy [12]. In an in vitro study, Ranzato et al. demonstrated that Manuka honey (0.1% v/v) increased the reepithelialization rate and keratinocyte chemoattraction in scratch wound and migration assays [16]. Kamaratos et al. reported that Manuka honey-impregnated dressings shortened the time required for neuropathic diabetic foot ulcers to heal [18]. Moreover, several wound dressings containing Manuka honey from New Zealand (Medihoney antibacterial honey Apinate dressings, Medihoney antibacterial honey gel sheets, and Medihoney antibacterial honey tulle–3 Ply dressings; Derma Sciences Europe Ltd., UK) have been used for wound care in the clinical setting in Europe and have been reported to be effective [18]. Therefore, we considered that the combined use of Manuka honey and HCD would have similar effects to wound dressings containing Manuka honey and promote cutaneous wound healing to a greater extent than the use of HCD alone. However, contrary to our expectations, the combined use of Manuka honey and HCD did not promote cutaneous wound healing to a greater degree than the use of HCD alone. For example, although the wound area of the Manuka + HCD group increased to the same extent as that of the HCD group and the wound area ratios of the Manuka + HCD and HCD groups did not differ significantly in the inflammatory phase, the wound area ratio of the Manuka + HCD group was significantly greater than that of the HCD group on days 8–14. Moreover, the reepithelialization and collagen fiber ratios of the Manuka + HCD group were significantly lower than those of the HCD group on days 7–14 and 11 and 14, respectively. These results show that the combined use of Manuka honey and HCD did not promote cutaneous wound healing compared with the use of HCD alone; in fact, it delayed cutaneous wound healing. We will attempt to clarify the reason for this in future.

## 5. Conclusion

It was demonstrated that the combined use of Japanese honey and HCD did not promote cutaneous wound healing compared with the use of HCD alone; in fact, it delayed cutaneous wound healing. Thus, this method is probably not useful for promoting cutaneous wound healing. Therefore, we plan to produce a wound dressing containing Japanese honey, as dressings containing Manuka honey have been demonstrated to be effective at promoting wound healing, and evaluate its effectiveness in the near future.
